# Eudaimonic and Hedonic Psychological Well-Being among Parents of Children with Cancer before and during the COVID-19 Pandemic: A Comparative Cross-Sectional Study

**DOI:** 10.3390/jcm11041113

**Published:** 2022-02-19

**Authors:** Natalia Ziółkowska, Kamilla Bargiel-Matusiewicz, Ewa Gruszczyńska

**Affiliations:** 1 Institute of Psychology, SWPS University of Social Sciences and Humanities, Chodakowska Street 19/31, 03-815 Warsaw, Poland; egruszczynska@swps.edu.pl; 2 Faculty of Psychology, University of Warsaw, Stawki Street 5/7, 00-183 Warsaw, Poland; kmatusiewicz@psych.uw.edu.pl

**Keywords:** COVID-19 pandemic, psychological distress, parents of children with cancer, psychological well-being, mental health, satisfaction in life, stress

## Abstract

The main aim of this study was to analyze the eudaimonic and hedonic well-being of parents of children with cancer by considering the role of a global stressor—the COVID-19 pandemic. One group of parents was assessed during the COVID-19 pandemic, and the other was assessed before it occurred. It was hypothesized that there may be a cumulative effect of stress, with parents expressing lower well-being during the pandemic due to the accumulation of stress related to their child’s illness and the pandemic. In total, 310 parents participated in the study. Following propensity score matching, 111 pairs were established. The results of the comparative analyses did not support the hypothesis, as the groups did not differ significantly in their reported levels of either eudaimonic (F (1.93) = 0.11, *p* = 0.75, η_p_^2^= 0.001) or hedonic well-being (F (1.100) = 0.02, *p* = 0.89, η_p_^2^ = 0.0001). These findings showed a limited effect of the COVID-19 pandemic on the well-being of parents of children with cancer. The oncological disease of a child is likely to be the central and the strongest factor for the parents, meaning that an additional, global stressor does not cause deeper exacerbation of their well-being.

## 1. Introduction

At the end of December 2019, a report appeared describing a group of patients suffering from pneumonia of unknown etiology in the city of Wuhan, China. This was followed by news of a possible link between the new coronavirus and the so-called wet market in Wuhan [1]. In late January 2020, in medical journal *The Lancet*, Ghinai, McPherson, et al., in the US, reported the first case of human-to-human transmission of the new coronavirus. In Europe, the first case of SARS-CoV-2 was reported in France in January 2020. On 11 March 2020, the World Health Organization (WHO) declared a global pandemic. Since then, SARS-CoV-2 and its associated disease, COVID-19, have seriously challenged the entire modern world [2]. In addition, the WHO has announced that the novel coronavirus and COVID-19 undoubtedly pose a threat to public health. 

In Poland, on 4 March 2020, the first case of SARS-CoV-2 was found in a patient returning from Germany [3]. The first “hard” lockdown was introduced in Poland on 16 March 2020, five days after the official announcement of the pandemic by the WHO. As of the date of this article’s submission, the statistics in Poland are as follows: since 4 March 2020, 5,519,282 people have been infected and 109,509 people have died. Initially, SARS-CoV-2 infections in children were much less common than in adults and, when infected, children had a much milder course of symptoms. However, in April 2020, there were reports regarding the connection between SARS-CoV-2 and pediatric inflammatory multisystem syndrome (PIMS), later called MIS-C syndrome [4]. Between 20 and 40% of the children diagnosed with COVID-19 met the criteria for Kawasaki disease, while the mortality rate for children with PIMS is estimated to be 2% [5]. It is worth noting that children with cancer are particularly vulnerable; therefore, for parents of children with oncological diseases, the numerous reports of the association between SARS-CoV-2 and MIS-C syndrome may be an additional source of stress. 

Relatively few studies have examined parents faced with a child’s oncological disease; however, globally, 300,000 children and teenagers under the age of 19 receive such a diagnosis each year and, unfortunately, cancer is one of the leading causes of death in this age group [6,7]. For parents, a child’s illness is one of the most significant possible sources of stress [8,9,10], strongly affecting their well-being. According to a definition widely used in psychology, “well-being refers to people’s evaluations of their lives—evaluations that are both affective and cognitive” [11] (p. 34). 

The understanding of well-being can be detailed by employing two philosophical systems: (1) hedonic and (2) eudaimonic. The concept of hedonism was first formulated by Aristippus of Cyrene, who claimed that happiness means experiencing sensual pleasure, and that a good life consists of maximizing such experiences [12]. This way of thinking was further developed by Epicurus, who asserted that pursuing the intensification of pleasure is the main goal in life [13]. Eudaimonism stands in contrast to hedonism; it was first described by Aristotle, who claimed that attaining real happiness requires finding and identifying one’s own virtues and then cultivating and developing them [13]. The eudaimonic attitude concerns the current emotions and experiences of a human being as they relate to self-realization [12,14]. A person aims at personal fulfillment in terms of developing their own virtues and individual potential which, in turn, promotes the realization of their own goals in life [12,15]. Eudaimonism understands well-being as seeing the sense of—and being certain of fulfilling—valuable goals. 

Ryff (1989) broadly reviewed the existing literature, and distinguished six factors in her concept of well-being; the author highlighted that the main strength of this model is its holistic view of well-being, going beyond a focus on the experience of positive emotions [14]. Therefore, well-being can also be treated as an indicator of adaptation to stressful situations in an individual’s life [16]. However, the research to date—both cross-sectional and longitudinal—has been dominated by a hedonic approach to well-being, i.e., an analysis of life satisfaction and affect. There is relatively little research on well-being in a eudaimonic context, especially in relation to specific stressful situations, even though such situations are known to trigger a search for meaning [17,18]. This points to the need for further exploration of this area, and the well-being of parents facing the serious illness of their child definitely needs to include a eudaimonic dimension in order to fully describe their functioning. 

Disease in a child—particularly chronic disease—undoubtedly constitutes a stressful situation for the child’s parents. This is an unforeseeable and extremely difficult event in the lives of the child’s parents, and possesses a high burdening potential [19]. The subjective well-being of parents of children diagnosed with an oncological disease is lower than that of control groups of parents of healthy children [20]. Additionally, a child’s oncological disease causes multiple changes in the parents’ lives, accompanied by strong emotions [21], leading to distress and disrupting the functioning of the whole family system [22]. Consequently, the prevalence of depression symptoms among the parents can be as high as 40% [23], and post-traumatic stress symptoms have been noted [24,25,26]. Thus, the majority of studies underline the severity of the parental distress during the oncological treatment of their child [27]; however, there are also studies—albeit mainly qualitative ones—indicating that the parents facing the serious disease of their child may change their life goals and redefine their purpose in life [28,29,30]. Such highly individual processes may be additionally modified by collective health-related stress [31,32,33].

Presently (during the COVID-19 pandemic), researchers have been focusing on the psychological functioning of the general population. In contrast, the research presented here concerns a clinical group for whom the main source of stress is the child’s oncological disease, but who also experience an additional stressor. Specifically, parents facing their child’s potentially fatal disease must additionally face the threats and consequences resulting from the COVID-19 pandemic, which is widespread, long lasting, and severe, and also characterized by unpredictability and uncontrollability—especially from an individual perspective. This defines the pandemic as a source of chronic stress. It is worth noting that the impact of the pandemic on parents of children with oncological illnesses can take several pathways: (1) concern for one’s own life and health in the context of COVID-19; (2) concern about their child becoming sick with COVID-19; (3) concern about the functioning of the health service, e.g., access to medical care, threatened treatment scheduling, and the possibility of visiting the child during hospitalization; (4) concern about the deterioration of economic conditions for the family; or (5) the need to care for the siblings of the child with the disease, or for other family members. For instance, a study of the parents of children with oncological illnesses during the COVID-19 pandemic [34] found that the vast majority of them were concerned about the SARS-CoV-2 virus and the possible transmission of the virus to their children. For 69.4% of the parents, the hospital was no longer a safe place. Additionally, the parents reported anxiety, as well as the psychological, social, and economic impacts of isolation [34].

Thus, a cumulative effect may occur resulting from the interaction of an individual and specific stressor—i.e., a child’s illness—and a collective and universal stressor, i.e., a pandemic. Such a context, to the best of our knowledge, has not yet been studied. Studies related to coping with somatic diseases tend to focus on highly individual approaches and omit any wider context; even antecedents in Lazarus and Folkman’s model [35] allow for placing this process in a broader context. Moreover, in our study, well-being is analyzed not only from the hedonic point of view [36], but also from the eudaimonic perspective [14], since according to the existing findings both life-threatening disease of a child and chronic stress due to the pandemic are likely to trigger search for meaning and meaning-focused coping [17,37,38]. 

The main aim of this study is to examine whether there is a cumulative effect of stress among parents of children diagnosed with cancer and undergoing medical treatment. Specifically, we hypothesize that there will be significant differences regarding both hedonic and eudaimonic well-being between two groups of parents: one assessed before the pandemic, and the other assessed during the pandemic. Consequently, the major difference between these groups is the difference in situational conditions, which is completely beyond the parents’ control.

## 2. Materials and Methods

### 2.1. Study Design

This is a comparative cross-sectional study, with the same specific condition in two groups (i.e., a child’s disease), but different unspecific conditions (i.e., the COVID-19 pandemic). According to the cumulative effect, well-being should be lower in the latter group, under the COVID-19 pandemic condition. 

### 2.2. Procedure and Participants

In total, 310 parents of children suffering from an oncological disease during active treatment participated in the study in two groups: 197 before and 113 during the COVID-19 pandemic. Considering the possible differences in confounding variables, propensity score matching was used to reduce the bias in comparisons between the two groups [39] (details in the Preliminary Results section). As a result, 111 pairs were established; among them were 111 parents whose children had undergone active stationary treatment in an oncology clinic before the COVID-19 pandemic, under regular conditions (Group 1, G1), and this group constituted the control group; another 111 parents took part in the study during the COVID-19 pandemic (Group 2, G2), and their children were treated in an oncology unit. During hospitalization a child was cared for mostly by one parent (72.1%); both parents were present in the case of 27.9%. Most parents declared that their place of permanent residence was more than 50 km from the hospital. More than 50% of parents had a university degree (57.2%), and most assessed their economic situation as average or below average (70.3%). The other sociodemographic and clinical characteristics of the analyzed groups are provided (details in Section 3), when describing details of the matching procedure between them.

It is also useful to consider broader characteristics in order to contextualize the reality of the participants. In Poland, the economy has shown considerable resilience to a pandemic; GDP exceeded pre-pandemic levels in December 2021, and is expected to grow by 5.2% in 2022. Unemployment in Poland also remains at a relatively low level, estimated at around 5%. In the latest (2020) Human Development Index (HDI), Poland ranks 35th among 50 countries. However, even if the economy is staying in relatively good shape, people’s mental health is not. In fact, some symptoms may develop or worsen over the course of the pandemic. Thus, this situation can be regarded as a common and significant source of chronic stress [40]. 

This study received approval from the institutional ethics committee (opinion no. 5/2019). The study was conducted in the following high-reference public medical centers: the Children’s Memorial Health Institute in Warsaw and the Medical University of Silesia’s Independent State Clinical Hospital No. 1 in Katowice (before the pandemic), and the Department of Oncology of the Children’s Memorial Health Institute in Warsaw (during the pandemic). 

The study was conducted from January 2019 to February 2020, before the pandemic. During the pandemic, the study was conducted as soon as possible after the lockdown, under a strict sanitary regime from July to September 2020. Earlier, due to safety measures, access for people other than employed staff was denied. Participants were recruited while on the ward with their child. The purpose of the study and the procedure were carefully explained and presented in order to minimize discomfort with participation. All of the participants provided informed consent. The study was conducted by the first author of this article. The average time to complete the questionnaires, all in paper and pencil format, was approximately 25–30 min. Participants were not compensated for their participation in the study.

### 2.3. Measures

The Psychological Well-Being Scale [14,41], as adapted by Krok (2009), was used to measure eudaimonic aspects of well-being; it consists of 42 statements, grouped in 6 subscales with 7 items each: (1) autonomy, which means independence and self-determination; (2) self-acceptance, which means accepting one’s own virtues and vices; (3) personal growth, meaning self-realization; (4) purpose in life, meaning having a sense of living and possessing a life goal; (5) environmental mastery, which means having a sense of influence; and (6) positive relations with others, which means possessing the ability to form satisfactory social relations [16,42]. The individuals assess each item on a 7-point scale from 1 = “definitely don’t agree” to 7 = “definitely agree”, and then the means are counted. Higher values point to a higher intensity of eudaimonic aspects of well-being. In the original version of the PWB, Cronbach’s α values for subscales ranged from 0.86 to 0.93 [14]; in the Polish adaptation these were from 0.72 to 0.86 [41]. 

The Satisfaction with Life Scale (SWLS) [43,44], as adapted by Juczyński (2009), was used to measure general satisfaction with life, which represents a cognitive aspect of hedonic well-being. The scale consists of 5 items describing general satisfaction with life. The participants mark their answers on a 7-point scale (from 1 = “strongly disagree” to 7 = “strongly agree”), and then the means are calculated. For the original version of the SWLS, the internal reliability coefficient was 0.87 [43], with a similar value of 0.81 for the Polish adaptation of the tool [44]. 

The Cronbach’s alphas obtained in the present study in both groups of parents (G1 and G2) are provided (details in the Section 3). 

### 2.4. Statistical Analyses

The propensity score matching procedure was used to match participants recruited before and during the COVID-19 pandemic with respect to their key relevant characteristics. Once matched, observations from before and during the COVID-19 pandemic were treated as dependent. Therefore, in order to compare levels of eudaimonic well-being and hedonic well-being, the within-subject designs MANCOVA and ANCOVA, respectively, were used. The SPSS version 26 program [45] was used to carry out all analyses. For PMS, the MatchIt package in R was used [46].

## 3. Preliminary Results

### Propensity Score Matching

Based on the literature [47], we identified the following key matching variables: respondent’s age, respondent’s sex, child’s age, and child’s sex. We used two of the most popular matching methods: exact matching, and nearest neighbor matching [48]. The exact matching procedure revealed only 10 pairs of observations, while nearest neighbor matching revealed 111 pairs; the results of the latter are depicted in Table 1.

For the next step, we conducted a series of analyses comparing the newly obtained G1 (n1 = 111) and G2 (n2 = 111). Specifically, using a series of chi-squared tests, we compared the distribution of education level, economic situation, hospitalization, type of cancer, stage of cancer, chemotherapy, radiotherapy, supportive treatment, having siblings, and being in a relationship. These analyses revealed significant differences in hospitalization (χ^2^ (1.218) = 60.04, *p* < 0.001) and the type of cancer (χ^2^ (1.219) = 12.71, *p* < 0.001). Therefore, these two variables will be entered as covariates into further analyses. Distributions of the other abovementioned variables did not differ significantly between the groups. The analysis showed that missing data present in the dependent variables are completely random (Little’s MCAR test: χ^2^ (1034) = 984.617, *p* = 0.862). For covariates, missing values constituted less than 5% of the sample (i.e., less than 10 observations). 

Key descriptive statistics of the outcome variable—eudaimonic well-being—are reported in Table 2. As indicated by skewness and kurtosis [49], the assumption of a normal distribution of dependent variables was met. The assumption of sphericity was violated for the eudaimonic well-being index in both its main and interaction effects; therefore, test values were reported with the Greenhouse–Geisser correction. For all other effects based on comparison of two levels, only the sphericity assumption does not apply [49].

## 4. Results

### 4.1. Eudaimonic Well-Being

**Figure 1:**
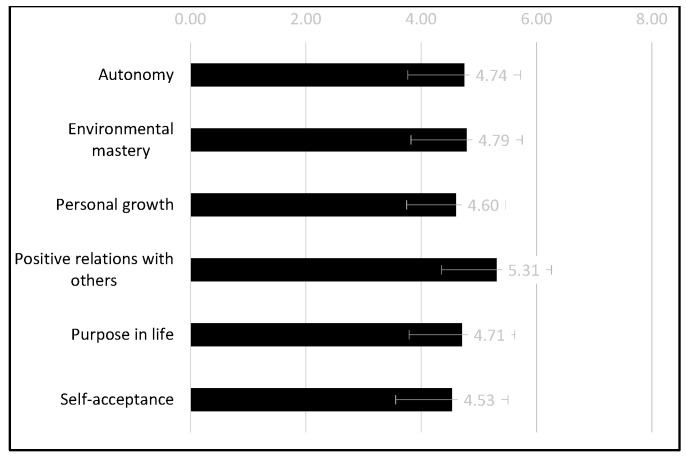
Mean and standard deviation of eudaimonic well-being types.

To test intergroup differences in six indices of eudaimonic well-being we conducted MANCOVA with condition—i.e., group (normal vs. pandemic)—as an independent variable, and with hospitalization and type of cancer as covariates. The main effect of the group was not significant (F (1.93) = 0.11, *p* = 0.75, η_p_^2^ = 0.001), and neither was the interaction of group x indices of eudaimonic well-being (F (5.465) = 1.10, *p* = 0.36, η_p_^2^= 0.01 with Greenhouse–Geisser correction). Only a significant main effect of the eudaimonic well-being index was observed (F (5.465) = 3.05, *p* = 0.01, η_p_^2^ = 0.03 with Greenhouse–Geisser correction). Figure 1 presents the means and standard deviations of all eudaimonic well-being indices. The mean of positive relations with others was significantly higher than the means of other subscales (*p* < 0.001). Self-acceptance was significantly lower than autonomy (*p* = 0.031), environmental mastery (*p* < 0.001), and purpose in life (*p* = 0.041). Personal growth was significantly lower than environmental mastery (*p* = 0.002). No other differences were significant.

### 4.2. Hedonic Well-Being

For satisfaction with life, we performed ANCOVA with the group as the independent variable, and adjusting for hospitalization and the type of cancer. The effect of being a parent of a child diagnosed with cancer in normal vs. pandemic conditions was insignificant (F (1.100) = 0.02, *p* = 0.89, η_p_^2^ = 0.0001). Thus, there were no significant differences in satisfaction with life between the two groups.

## 5. Discussion

The main aim of this article was to examine eudaimonic and hedonic well-being between two groups of parents of children suffering from oncological disease, under different measurement conditions. Mainly, half of the parents were assessed before the COVID-19 pandemic, whereas the other half were assessed during the pandemic. The cumulative effect of stress was expected, with parents under the pandemic conditions expressing lower well-being due to the build-up of their children’s illness-related stress and pandemic-related stress. However, this hypothesis was not supported—the groups reported the same levels of both eudaimonic and hedonic well-being. This is an interesting result, particularly as studies conducted among the general population have shown that the COVID-19 pandemic is associated with decreased well-being. However, this deterioration of well-being is operationalized in negative terms only, as an increase in anxiety and depression symptoms [50,51,52,53]. 

A study conducted in Italy among the general population during a nationwide lockdown found that the relationship between life satisfaction and perceived stress is partially mediated by coping and positive attitudes [54]. Another study of Brazilian students found that most of the participants had high levels of anxiety, depression, and distress. Moreover, these symptoms were negatively related to all dimensions of psychological well-being in this group. In addition, predictors of depression, anxiety, and distress included gender (female), age, chronic disease burden, and lower scores on two dimensions of eudaimonic well-being (positive relationships with others and self-acceptance) [55]. Another study, conducted in the UK, found that levels of depression tested before and during the COVID-19 pandemic did not differ between the study groups, while anxiety levels increased significantly during the pandemic [56]. Satisfaction with life has been measured in a limited number of studies, with perceived social isolation having a negative impact [57]. Moreover, in studies using quality of life as an index describing broader aspects of functioning, the results are inconclusive. For instance, a Danish study of adult oncology patients found no significant differences in quality of life or emotional functioning compared to the Danish “Barometer Study”; however, fear of SARS-CoV-2 infection was correlated with lower quality of life in this group [58]. Furthermore, a study conducted in Australia among people with type 2 diabetes found a negative impact of the pandemic on quality of life, related to age of the participants—younger people reported lower quality of life; anxiety and depressive symptoms remained relatively unchanged [59]. This may suggest different effects of the pandemic for negative and positive components of well-being. In this respect, in our study, we found no additional effect of the COVID-19 pandemic on positively defined well-being among people who were already under stress due to an individual condition.

According to the theoretical framework and empirical findings regarding well-being [16,60,61], relatively constant characteristics of an individual over the long term—although sensitive to life events—may have positive or negative potential. However, in general, this effect is only short term, as individuals tend to return to what is known as the “set point”, or baseline position [16,62,63,64]. This has been extensively studied in the case of hedonic well-being, with fewer data on eudaimonic well-being, where more permanent quantitative and qualitative changes may have occurred under the influence of critical life events.

However, there is a research gap regarding the well-being of parents of children with cancer. Furthermore, there have been few studies in this area, and none has considered the cumulative source of stress. However, the studies that do exist indicate that the subjective well-being of parents of children with oncological disease is often lower compared to control groups of parents of healthy children [20,65]. On the other hand, other research indicates that parents of children with cancer have higher satisfaction in life than parents of healthy children, despite high levels of anxiety and depression in this group [66]. Therefore, in this group, positive and negative dimensions of well-being should be assessed, as they may be relatively independent. It can be assumed, when attempting to understand why there were no significant differences related to the COVID-19 pandemic in the well-being of groups of parents of children with oncological illnesses examined before and during the pandemic, that the influence and role of oncological disease as a source of stress are likely so strong that additional stressors do not play a significant role. Thus, it is not a cumulative effect observed here, but rather a removal of the effect of more general stressors. As mentioned in the Introduction, a child’s illness is one of the most significant possible sources of stress [8,9,10]. Experiencing a threat to a child’s life, in the form of an oncological disease, violates one of the most basic human needs, i.e., the need to feel safe for oneself and for those closest [67,68]; it is also an existential threat, because it is a new situation that appears unexpectedly. A sense of anxiety, hopelessness, and helplessness causes questions to be asked regarding one’s own sense of agency and worth. Thus, it is highly likely that the child’s illness becomes the central stressor in the parent’s life, having the greatest impact on the parent’s well-being, as in the figure–ground principle.

However, our study has some limitations, which should be considered when discussing the results. Firstly, this study was cross-sectional, preventing the formulation of conclusions regarding causality. In future research, a longitudinal study should be conducted, despite difficulties that involve access to the parents and their willingness to participate, which also raise ethical issues—especially since the death of a child cannot be ruled out. Secondly, the study was limited not only by the relatively small number of participants, but also by their gender distribution. The vast majority of participants were mothers of sick children. This sampling imbalance was because mothers are usually the primary caregivers both in and outside of the hospital [69]; furthermore, the strict sanitary regime prevented two-parent care. Another limitation revolves around differences in access to groups of parents in hospitals as a result of pandemic restrictions. Specifically, before the pandemic, the study was conducted in a stationary oncology clinic and chemotherapy day unit, whereas during the pandemic the only available option was assessment at a chemotherapy day unit, where children were treated during short hospital stays. Finally, this study did not include negative measures of well-being—namely, depression and anxiety—and only the cognitive aspect of hedonic well-being was measured. Furthermore, relatively low internal consistency of two of the subscales (i.e., personal growth and purpose in life) in the group evaluated before the pandemic was found. Additionally, the lack of inclusion of parents of healthy children made it impossible to compare the well-being of parents of sick children with a group experiencing only a universal health-related stressor—namely, the pandemic, which affects the entire population. Future research should assess parents’ well-being more broadly and under more varied conditions.

Overall, in our study—contrary to expectations—the COVID-19 pandemic did not translate into a further reduction in the well-being of parents of children suffering from an oncological disease, which may be explained by the centrality and seriousness of their major source of stress. The obtained results, therefore, suggest that when stressors are co-occurring, their effect may depend on the individual importance assigned to them. This supports Lazarus and Folkman’s [35] assumption regarding the role of a subjective cognitive appraisal in shaping the stress response and coping process. However, the area of stressor interaction, despite its high ecological validity, remains virtually unexplored. Moreover, as our study was cross-sectional, the findings should be treated with caution, and further research is needed. 

## 6. Conclusions

This study has two major contributions: From the theoretical point of view, thanks to the adopted methodological design, we tested well-being with systematical identification of two sources of stress: a child’s illness, and the COVID-19 pandemic. We found that co-existence of these two sources did not translate into worse well-being of the parents of children with cancer. We proposed an interpretative framework assuming that different sources of stress have different effects on well-being, depending on their centrality and importance to the individual. Thus, the final effect may not be the result of an objective accumulation of stressors, but rather of a figure–background mechanism of giving leading meaning to one of them. Future research is needed in order to identify this coping mechanism of dynamically assigning and/or withdrawing personal meaning to/from co-occurring and chronic stressful situations.

The practical value of this study lies in the fact that stress experienced by parents—and, consequently, changes in their well-being—may be crucial not only for the psychological comfort of diagnosed children, but also for cooperation with medical personnel, which can ultimately affect the treatment process [26]. Thus, parents often need and require professional psychological help. Despite that, the lack of adequate empirical research is a major obstacle to the development of evidence-based programs tailored to these needs. Thus, this study inspires further research with potential clinical implications for effective psychological support of primary caregivers of children with severe somatic illnesses.

## Figures and Tables

**Table 1:** Summary of balance for matching variables.

Variable	Group 1		Group 2			
	M	SD	M	SD	SMD	*p*-Value
	Before Matching					
**Parent’s age**	**36.31**	**6.45**	38.0	6.41	0.26	0.028
Parent’s sex	0.22	0.41	0.20	0.40	0.04	0.761
Child’s age (months)	86.60	65.72	97.35	53.08	0.09	0.142
Child’s sex	0.63	0.48	0.58	0.50	0.18	0.432
	**After Matching**					
Parent’s age	37.77	5.91	38.05	6.42	0.05	0.736
Parent’s sex	0.20	0.40	0.21	0.41	0.02	0.868
Child’s age (months)	97.97	68.60	0.59	0.49	0.04	0.786
Child’s sex	0.58	0.50	97.35	53.08	0.01	0.941

Note: Sex coded as: 0—woman, girl; 1—man, boy; M—mean; SD—standard deviation; SMD—standardized mean difference; Group 1—parents during regular conditions; Group 2—parents during the pandemic.

**Table 2:** Descriptive statistics of well-being.

Variable	Group 1				Group 2				Cronbach’s α Group 1	Cronbach’s Group 2
	M	SD	Skewness	Kurtosis	M	SD	Skewness	Kurtosis		
Autonomy	4.47	0.96	−0.06	0.29	4.75	0.99	0.001	−0.67	0.76	0.77
Environmental mastery	4.80	1.02	−1.09	1.69	4.77	0.91	−0.05	−0.47	0.83	0.77
Personal growth	4.53	0.75	0.07	−0.26	4.67	0.94	−0.04	−0.03	0.56	0.75
Positive relations with others	5.25	0.97	−0.57	1.06	5.36	0.94	−0.21	−0.73	0.79	0.81
Purpose in life	4.68	0.85	−0.31	0.22	4.74	0.97	0.16	−0.52	0.59	0.73
Self-acceptance	4.44	0.98	−0.42	0.84	4.61	0.97	−0.22	0.25	0.78	0.81
Hedonic well-being	4.02	1.14	−0.09	−0.37	4.26	0.99	−0.27	−0.42	0.83	0.78

Note: M—mean; SD—standard deviation; *α*—Cronbach’s α.

## Data Availability

The presented data in this study are available from the corresponding author upon reasonable request.
